# SIEDY Diagnostic Accuracy in Assessing Erectile Dysfunction in Young Men Living With HIV

**DOI:** 10.1111/andr.70215

**Published:** 2026-03-09

**Authors:** Giorgio Tiecco, Federico Cesanelli, Cosimo Colangelo, Marco Di Gregorio, Andrea Delbarba, Paolo Facondo, Matteo Riva, Carlo Cappelli, Emanuele Focà, Francesco Castelli, Eugenia Quiros‐Roldan

**Affiliations:** ^1^ Department of Clinical and Experimental Sciences Unit of Infectious and Tropical Diseases University of Brescia and ASST Spedali Civili di Brescia Brescia Italy; ^2^ Department of Clinical and Experimental Sciences Unit of Endocrinology and Metabolism University of Brescia and ASST Spedali Civili di Brescia Brescia Italy; ^3^ Department of Medical and Surgical Specialties Radiological Sciences and Public Health University of Brescia ASST Spedali Civili of Brescia Piazzale Spedali Civili Brescia Italy; ^4^ Department of Global Public Health Karolinska Institutet Stockholm Sweden

**Keywords:** dynamic penile color doppler ultrasound, erectile dysfunction, HIV, IMT, sexual dysfunction, SIEDY

## Abstract

**Background:**

Erectile dysfunction (ED) is a multidimensional condition commonly affecting men living with HIV. The structured interview of erectile dysfunction (SIEDY) is the only validated tool assessing organic, relational, and psychological factors contributing to ED, while dynamic penile color Doppler ultrasound (dPCDU) is a reliable method for evaluating vascular causes of ED.

**Objectives:**

This monocentric cross‐sectional study aims to assess the reliability of the SIEDY questionnaire in determining the underlying causes of ED in young men living with HIV.

**Materials and Methods:**

Young men (<50 years) living with HIV, on antiretroviral therapy for at least the last 12 months, and reporting symptoms of ED were enrolled. The degree of sexual dysfunction was assessed with the International Index of Erectile Function‐5 (IIEF‐5), while SIEDY was used to explore the pathogenetic components of ED. dPCDU was performed to assess vascular damage in the penile district. Univariate binomial logistic regression models were used to evaluate the association between ultrasound parameters and SIEDY results.

**Results:**

A total of 50 young men living with HIV were enrolled, of whom 40 (80%) had at least one altered SIEDY scale, most frequently Scale 3 (70%). Forty‐five (90%) participants presented with at least one abnormal ultrasound parameter, with intima‐media thickness being the most common (76%). No correlation was found between ultrasound parameters and the SIEDY Scale 1, while logistic regression identified IMT as the only variable significantly associated with SIEDY Scales 2 (relational) and 3 (psychological).

**Discussion and Conclusions:**

SIEDY may have limited reliability in detecting vascular causes of ED in this population, particularly underestimating organic causes. The lack of correlation with objective vascular findings highlights the importance of including tools like dPCDU for vascular assessment in routine clinical evaluations of the ED.

## 1 Introduction

In recent decades, advances in efficacy and accessibility of HIV testing and antiretroviral therapy (ART) have totally changed the natural history of HIV infection, with an increase in life expectancy that is now approaching that of the general population [1, 2]. However, success in the fight against HIV cannot be measured only by biological parameters, such as viral load suppression, but must also account for the overall well‐being of people living with the virus (PLWH). Therefore, to expand the UNAIDS 2025 targets, it has already been suggested to add a new “fourth 95” goal to ensure that 95% of people with viral load suppression achieve good health‐related quality of life (HRQoL) [3, 4].

Sexual dysfunction is a comorbidity that strongly affects HRQoL [5], and it is frequently described among PLWH [6, 7, 8, 9]. Erectile dysfunction (ED) is a common condition in older men, and it is defined as the inability to obtain or maintain a satisfactory erection for sexual intercourse [9, 10]. The prevalence of this condition is higher, and it has an earlier onset in PLWH compared with the general population [6, 11]. Recent studies have reported strikingly high prevalence rates in PLWH, including 73.8% in a cohort with a mean age of 40.4 ± 12.4 years in Western Turkey [12] and 74.6% in a population with a median age of 50 years in Northern Tanzania [13]. There are several factors that may contribute to the pathogenesis of ED in young men living with HIV (yMLWH), such as the infection itself, ART, comorbidities, or psychological issues related to the infection (e.g., stigma, fear of infection, low body satisfaction) [6, 9, 14, 15]. However, the pathogenetic mechanisms are still largely unknown, as the specific weight of every etiological factor, including organic, metabolic, hormonal, inflammatory, and psychological factors, remains unclear.

The most recent European AIDS Clinical Society (EACS) [16] guidelines for management of PLWH recommend assessing ED through a self‐administered questionnaire (International Index of Erectile Function—IIEF), which determines not only the presence of the condition but also its severity and its impact on sexual life, although it does not provide insights into the underlying etiology of the condition [17, 18, 19]. The structured interview on erectile dysfunction (SIEDY) is another validated questionnaire aimed at assessing the etiologic factors contributing to ED [20, 21, 22]. Recently, SIEDY has been utilized in yMLWH, highlighting psychological factors as the most frequently associated components in the onset of ED within this population [23]. Although SIEDY is recommended in certain clinical guidelines, its sensitivity and specificity are relatively poor and vary depending on the domain assessed [24]. Specifically, the SIEDY Scale 1, which evaluates the organic component of ED, demonstrates a sensitivity and specificity of 68% [20]. SIEDY Scale 2, which assesses the relational aspects (for instance, marital impairment), exhibits a sensitivity of 53% and a specificity of 66% [21], while SIEDY Scale 3, which predicts psychopathology, has a sensitivity of 76% and a specificity of 61% [22].

Another diagnostically relevant tool for ED is dynamic penile color Doppler echography (dPCDU), as highlighted in the latest Andrology guidelines [24, 25, 26, 27, 28]. This technique is a well‐established and reliable procedure, widely recognized as the gold standard for assessing penile vascular pathologies and their severity. dPCDU is a safe, low‐risk diagnostic test with complications occurring infrequently [29]. Its application may be particularly valuable for men requiring accurate cardiovascular risk stratification, including PLWH, as it facilitates the early identification of individuals who may benefit from comprehensive cardiovascular assessment [30, 31]. However, the specific role of vascular factors in ED among PLWH remains poorly investigated in the existing literature.

This monocentric cross‐sectional study aims to assess the reliability of the SIEDY questionnaire in determining the etiology of ED in yMLWH.

## 2 Methods

### 2.1 Study Design and Participants

This monocentric cross‐sectional study enrolled yMLWH experiencing ED who attended the Unit of Infectious and Tropical Diseases at the Spedali Civili Hospital of Brescia, Italy, between June and December 2023. All yMLWH were systematically screened for ED symptoms during routine follow‐up visits. Eligibility criteria included a confirmed HIV diagnosis, a minimum of 12 months on ART, and an age range of 18 to 50 years, with the latter restriction implemented to minimize the influence of age‐related factors on ED. No exclusion criteria were considered. Finally, only the yMLWH reporting EDs were included in the analysis. These participants underwent a comprehensive multidisciplinary assessment, which encompassed a specialist consultation in endocrinology and andrology from ASST Spedali Civili di Brescia, along with the administration of the SIEDY and IIEF questionnaires and dPCDU.

### 2.2 Sexual Function Assessment

In case of referred ED, sexual function was assessed through two validated questionnaires: IIEF‐5 and SIEDY. The IIEF is a validated, multidimensional questionnaire approved by the National Institutes of Health (NIH) to assess five key domains of sexual function: erectile function, orgasmic function, sexual desire, satisfaction with intercourse, and overall sexual satisfaction [17, 18]. The IIEF‐5 (5‐item version specific for ED) is recommended for evaluating sexual dysfunction in all MLWH thanks to its accuracy that makes it particularly practical for clinical use. IIEF‐5 scores range from 5 to 25, and ED severity is classified into five categories: severe [5, 6, 7], moderate [8, 9, 10, 11], mild to moderate [12, 13, 14, 15, 16], mild [17, 18, 19, 20, 21], and normal [22, 23, 24, 25]. The questionnaire was not administered to individuals who had not engaged in sexual intercourse within the past 6 months [17, 18]. The SIEDY is a multidimensional validated questionnaire for the pathogenetic assessment of ED, evaluating three domains of ED pathophysiology (organic, marital, and intrapsychic) that frequently coexist in its development [20, 21, 22]. The interview consists of 13 questions and 2 ancillary ones that the physician must administer to the patient. Each question involves answers with a pre‐coded score (0–3). The questions administered are part of three groups called scales: Scale 1 (3 questions for 9 points) assesses the organic component; Scale 2 (4 questions for 12 points) evaluates the relational component; Scale 3 (6 questions for 18 points) explores the psychogenic component of ED. A Scale 1 score ≥ 3.5 indicates the presence of an organic component underlying ED, whereas scores ≥ 2 on Scale 2 and ≥ 3 on Scale 3 suggest marital impairment and psychopathology, respectively [20, 21, 22].

### 2.3 Data Collection

During the multidisciplinary visit, we collected demographic data and anthropometric measurements (weight, height, and body mass index [BMI]). A comprehensive physical examination was conducted, and vital signs were recorded. Participants were interviewed regarding their past medical history, smoking habits, and any prior use of phosphodiesterase 5 inhibitors (IPDE‐5). Comorbidities were identified according to the last version of EACS guidelines [16]. Data on sexual orientation were extracted from electronic clinical records as recorded at the first Infectious Disease visit of the PLWH included. Questionnaires were administered by an experienced andrologist. Collected data, including blood samples (counts, metabolic, and hormonal assays), are fully explained elsewhere [32].

### 2.4 Penile Doppler Ultrasonography

All patients reporting ED underwent dPCDU, performed by a single experienced specialist in endocrinology and andrology at ASST Spedali Civili di Brescia using high‐resolution, high‐frequency linear probes (7.5–14 MHz). The procedure began with an intracavernous injection of 10 µg alprostadil to induce an erection, followed by assessment of intracavernous blood flow at the peno‐scrotal junction over a 20‐min period. The key parameters measured were peak systolic velocity (PSV) and intima‐media thickness (IMT) of the right and left cavernous arteries to assess for ED secondary to arterial disease [24, 25, 26, 32, 33]. Arterial dysfunction was defined as a cavernous artery PSV < 35 cm/s or IMT ≥ 0.3 mm in at least one artery [34]. Veno‐occlusive dysfunction was identified when the end‐diastolic velocity (EDV) of the two arteries exceeded 5 cm/s or when the resistance index (RI) was < 0.9 [24, 33].

### 2.5 Ethics

The study protocol was approved by the Spedali Civili Ethics Committee (protocol no. 5184) and conducted in accordance with the ethical standards of the Helsinki Declaration (1975, revised in 2013). Written informed consent has been obtained from each subject.

### 2.6 Statistical Analysis

The mean values with standard deviation (SD) were used to describe continuous variables, while the counts and percentages were employed for categorical variables. The relationship between clinical variables and ultrasound parameters (categorical and/or continuous) was evaluated using the statistical method appropriate for their distribution. Regarding continuous variables, normality was tested using the Shapiro–Wilk test. If both groups followed a normal distribution, a Levene's test was performed to assess the homogeneity of variances. Based on the results, either a Student's *t*‐test (for equal variances) or Welch's *t*‐test (for unequal variances) was applied. If the distribution was not normal in at least one group, a Mann–Whitney *U* test was used instead. To explore associations between categorical variables, the Chi‐square test was used when all expected frequencies were greater than five; otherwise, Fisher's exact test was applied. When comparing the prevalence of pathological SIEDY scales within the same individuals, Cochran's Q test was applied to account for the non‐independence of related proportions; when significant, post‐hoc pairwise comparisons were performed using McNemar tests with Bonferroni correction. This analysis incorporated IMT and PSV, continuous or categorized as either physiological or pathological, with a threshold defined as IMT ≥ 0.3 mm and PSV < 35 cm/s. All dPCDU parameters were expressed as the mean of right and left values when considered as continuous variables; when considered as categorical variables, the presence of at least one pathological value on either side was sufficient to define the parameter as pathological. To evaluate the association between ultrasound parameters and questionnaire results, univariate binomial logistic regression models were fitted, considering the questionnaires as dependent variables and performing a *Z*‐test. Odds ratios (OR) and 95% confidence intervals (CI) were reported for each model. Statistical significance was set at *p* < 0.05. The analyses were performed using R and Jamovi statistical software [35, 36].

## 3 Results

After screening for eligibility, 310 patients fulfilled the inclusion criteria, among them 50 yMLWH reported ED and were enrolled. The participants had a mean age of 44.4 years (SD ±4.3), and 24 (48%) declared exclusive male‐to‐male sexual intercourses. All included individuals were virologically suppressed, with a mean CD4/CD8 ratio of 0.9 ± 0.45; 45 (90%) were on an antiretroviral regimen based on integrase inhibitors, and 27 (27/50, 54%) were on dual therapy. Hypogonadism was identified in 6 (11.8%) yMLWH. The most frequently reported comorbidities were arterial hypertension (10/50, 20 %) and dyslipidaemia (9/50, 18.0%). A detailed overview of the demographic and clinical characteristics of the cohort is available in detail elsewhere [32] and in Tables S1 and S2.

All participants were sexually active, with at least one intercourse in the preceding 6 months. An altered IIEF‐5 score was observed in 47 (94%) yMLWH, including 14 (28%) classified as having moderate to severe ED. At least one abnormal SIEDY scale was identified in 40 (80%) yMLWH, most commonly Scale 3 (35/50, 70%). Pathological findings on ≥ 2 SIEDY scales were present in 21 (42%) participants, whereas abnormalities on Scale 1 were observed in 14 (24%) yMLWH (concordance analysis among IIEF‐5 and SIEDY Scale 1, and statistical comparison among prevalences of pathological SIEDY scales are shown in Tables S3 and S4, respectively). Detailed questionnaire results are presented in Table 1.

**TABLE 1:** IIEF‐5 and SIEDY questionnaires results.

Sample size, *n* (%)	50 (100)
IIEF‐5	
Mild ED, *n* (%)	17 (34)
Mild to moderate ED, *n* (%)	16 (32)
Moderate ED, *n* (%)	6 (12)
Severe ED, *n* (%)	8 (16)
SIEDY	
Pathological scale 1, *n* (%)	14 (24)
Pathological scale 2, *n* (%)	18 (36)
Pathological scale 3, *n* (%)	35 (70)
At least one pathological scale, *n* (%)	40 (80)
Three pathological scales, *n* (%)	6 (12)
Pathological scales 1 and 2, *n* (%)	**0 (0)**
Pathological scales 1 and 3, *n* (%)	6 (12)
Pathological scales 2 and 3, *n* (%)	9 (18)

**FIGURE 1:**
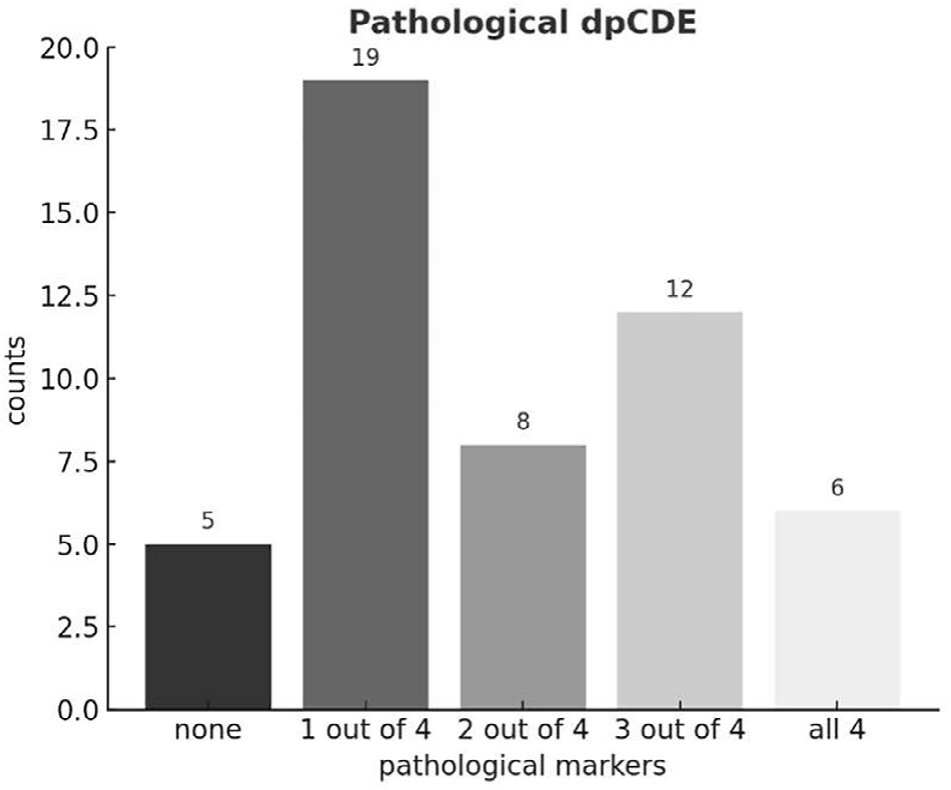
Number of pathological dPCDU markers.

Results from dPCDU assessments are detailed in Table 2 and Figure 1. Thirty‐eight (76%) yMLWH had a pathological IMT with a mean value of 0.307 ± 0.076 mm, and 10 (20%) had a pathological PSV with a mean value of 46.5 ± 16.1 cm/s. Forty‐five (90%) had at least one abnormal ultrasound marker. Among the five (10%) individuals with normal IMT, PSV, EDV, and RI, anatomical alterations of the cavernous arteries were identified in three (60%) cases. Six (12%) demonstrated pathological findings across all four ultrasound parameters.

**TABLE 2:** Dynamic penile color doppler ultrasound results.

Sample size, *n* (%)	50 (100)
Pathological response to injection, *n* (%)	9 (18)
Tardive response to injection, *n* (%)	8 (48)
Pathological IMT, *n* (%)	38 (76)
Pathological PSV, *n* (%)	10 (20)
Pathological EDV, *n* (%)	26 (52)
Pathological RI, *n* (%)	24 (16)
IMT, mean (SD)	0.307 (0.076)
PSV, mean (SD)	46.5 (16.1)
EDV, mean (SD)	4.4 (4.1)
RI, mean (SD)	0.882 (0.1)
Cavernous artery tortuosity, *n* (%)	15 (30)
Presence of arterial anastomosis, *n* (%)	25 (50)
Accessory cavernous arteries, *n* (%)	5 (10)
Penile plaques, *n* (%)	2 (4)

Abbreviations: EDV, end diastolic velocity; IMT, intima‐media thickness; PSV, peak systolic velocity; RI, resistance index.

**FIGURE 2:**
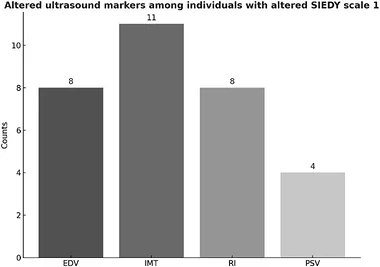
Altered ultrasound markers among individuals with altered SIEDY Scale 1. EDV, end diastolic velocity; IMT, intima‐media thickness; PSV, peak systolic velocity; RI, resistivity index.

Among the 38 people with an altered IMT, only 11 also had an altered SIEDY Scale 1. Out of the 14 having altered SIEDY Scale 1, only 3 had a normal IMT. The distribution of the ultrasound alterations among yMLWH with an altered SIEDY Scale 1 is shown in Figure 2.

We aimed to evaluate potential correlations between SIEDY Scales 1, 2, and 3 (considered as dichotomous categorical variables “pathological/not pathological”) and the ultrasound parameters IMT, PSV, EDV, and RI (continuous variables). When analyzing ultrasound variables in relation to the SIEDY Scale 1, none showed statistically significant differences. For SIEDY Scales 2 and 3, only IMT reached statistical significance (*p* = 0.029 and 0.042, respectively). When the ultrasound parameters were analyzed as categorical variables, no significant associations were found with any of the SIEDY scales.

Using univariate logistic regression models for SIEDY Scale 1, RI was the only significant predictor (*p* = 0.026), while other ultrasound variables did not reach statistical significance. IMT was the only variable that showed a statistically significant association with SIEDY Scales 2 and 3 (*p* = 0.031 and 0.045, respectively). No significant associations were observed between the other ultrasound parameters (PSV, EDV, RI) and SIEDY Scales 2 and 3.

## 4 Discussion

In this cross‐sectional, single‐center study, out of the 50 yMLWH who reported symptoms of ED, 47 (47/50, 94%) had at least one altered SIEDY scale, and 45 (45/50, 90%) had at least one alteration in dPCDU. SIEDY Scale 3 (psychological) was the most frequently altered; however, no correlation was found between ultrasound parameters and SIEDY Scale 1 (organic), while logistic regression identified IMT as the only variable significantly associated with SIEDY Scales 2 (relational) and 3 (psychological). These findings suggest that, although the SIEDY questionnaire provides insights into the potential contributing factors of ED, it does not appear to be a reliable tool for defining the etiology of ED in the yMLWH population.

The SIEDY questionnaire has been rarely used in the scientific literature after its validation because of its low accuracy, particularly in non‐organic pathogenetic scales [20, 21, 22]. However, it remains the only validated tool capable of simultaneously identifying and quantifying several pathogenetic domains of ED. Moreover, it is endorsed by the guidelines for ED issued by the Italian Society of Andrology and Sexual Medicine [24], and its applicability has already been evaluated in a sample of PLWH [23]. In our study, the SIEDY questionnaire revealed a high prevalence of the psychological and relational factors as causes of ED among yMLWH. SIEDY Scale 3 was the most frequently altered domain (70% of participants), followed by Scale 2 (36%), while Scale 1 was affected in only 24%. These results suggest that non‐organic factors play a predominant role in ED within this population, aligning with previous research highlighting the impact of psychological factors in PLWH [23]. However, despite this evidence, psychological and relational components are often overlooked in yMLWH reporting ED symptoms.

When comparing SIEDY results with dPCDU findings, we observed a significant discrepancy in identifying the organic component of ED. While only 24% of participants had an altered SIEDY Scale 1, 90% exhibited at least one altered penile ultrasound marker, with 70% showing pathological IMT values. This suggests that vascular alterations are more prevalent than indicated by the SIEDY 1 questionnaire, which might not accurately detect the organic component of ED. Statistical analyses further support this discrepancy, as in logistic regression models, IMT was a significant predictor only for SIEDY Scales 2 and 3. These results reinforce the hypothesis that SIEDY may have a poor ability to capture the organic component of ED in yMLWH. This limitation carries important clinical implications because, according to the 2024 Princeton IV consensus guidelines, the vasculogenic component of ED should always be carefully evaluated [37]. Indeed, vasculogenic ED alone constitutes an indication for coronary artery calcium scoring by computed tomography in men aged 40–60 years, regardless of prior cardiac history, even in the presence of only intermediate cardiovascular risk [37].

Although the study design does not allow for establishing a cause‐and‐effect relationship, the observed correlation between pathological IMT and SIEDY Scales 2 and 3 suggests that psychological and relational issues may mask underlying vascular damage in several patients [38, 39], warranting further investigations. A bidirectional association between psychiatric disorders and sexual dysfunction is well‐documented in HIV‐uninfected people. However, no published studies have directly linked ultrasound markers of penile vascular damage with a psychological index [40, 41].

Our findings also highlight the importance of incorporating objective diagnostic tools, such as dPCDU, in the assessment of ED in yMLWH. The high prevalence of vascular abnormalities in our cohort of young patients suggests that relying solely on self‐reported questionnaires may lead to an underestimation of the organic component of ED. This has critical clinical implications, as vascular ED is not only indicative of endothelial dysfunction in the penile district but also an early predictor of cardiovascular disease [42, 43]. Among ultrasound markers, PSV is commonly used for evaluating penile arterial function, while IMT is widely applied to assess atherosclerosis and vascular damage in carotid arteries [44]. Some studies have validated IMT as a predictor of ED in cavernous arteries as well [34, 45], suggesting it may provide a more accurate indication of vascular ED than PSV. Additionally, pathological IMT has been proposed as a sensitive predictor for systemic atherosclerosis [34, 40, 44]. In our sample, 38 (76%) individuals exhibited pathological IMT, confirming the high prevalence of this altered marker even in yMLWH with ED symptoms [46]. Nevertheless, the relationship between ED and systemic vascular damage in PLWH is not consistently supported by the literature. Some studies suggest that, in this population, ED may be more strongly associated with factors such as age and body image dissatisfaction rather than endothelial dysfunction [47, 48].

This study has strengths and limitations. The SIEDY questionnaire, while validated for assessing the multidimensional components of ED, has not specifically been validated for yMLWH, a population with specific psychosocial and biological characteristics. Furthermore, the low accuracy of the SIEDY questionnaire, particularly Scales 2 and 3, suggests that its results may be prone to misinterpretation. Moreover, both the IIEF‐5 and the SIEDY questionnaires are primarily validated in heterosexual men [17, 18, 20, 21, 22], while it is known that the yMLWH population is composed of people with more diverse sexual orientations. Nevertheless, at the first Infectious Disease visit, 52% of the included participants did not report an exclusive male‐to‐male sexual activity. Is type of investigation has not been conducted before; therefore, no formal sample size calculation was performed, and this study should be considered as a pilot. Finally, causal inference could not be performed because of the absence of a control group, which was not enrolled because of the difficulty in finding healthy young men eligible for a dPCDU. A key strength of this study is that, to the best of our knowledge, it is the first to comprehensively evaluate ED in yMLWH with both validated questionnaires (IIEF‐5 and SIEDY) and dPCDU. Furthermore, although PSV is considered the standard marker for penile arterial disease and is included in the Italian Society of Andrology and Sexual Medicine guidelines [24, 49], this is among the first studies to assess cavernous artery IMT in yMLWH. This parameter may provide greater accuracy in younger individuals, as it has shown superior sensitivity for early systemic atherosclerosis compared to PSV [34, 50], particularly in men under 50, such as those in our cohort. These findings may encourage further research into the relationship between cavernous artery IMT, psychological factors, and early cardiovascular pathology in yMLWH and other populations.

## 5 Conclusions

ED is a multifactorial condition in the general population, yet the interplay of contributing factors among yMLWH remains incompletely understood. Our findings suggest that the SIEDY questionnaire may not be a reliable tool for determining the etiology of ED in this population, particularly in identifying organic components. Our findings support the inclusion of dPCDU, including IMT measurement, in selected yMLWH presenting with ED, particularly when vascular causes are suspected. Because penile vascular alterations may precede systemic vascular dysfunction, early detection in yMLWH is of critical importance for broader cardiovascular risk assessment and monitoring.

## Author Contributions

Giorgio Tiecco, Andrea Delbarba, and Eugenia Quiros‐Roldan contributed to the study conception and design. Material preparation, data collection, and analysis were performed by Giorgio Tiecco, Federico Cesanelli, Cosimo Colangelo, Marco Di Gregorio, Andrea Delbarba, Matteo Riva, and Eugenia Quiros‐Roldan. The first draft of the manuscript was written by Giorgio Tiecco, Federico Cesanelli, and Eugenia Quiros‐Roldan. All authors read and approved the final manuscript.

## Funding

The authors have nothing to report.

## Ethics Statement

The study protocol was approved by the ASST Spedali Civili Ethics Committee (protocol NP 5184).

## Conflicts of Interest

The authors declare no conflicts of interest.

## Supporting information




**Supporting Table 1**: Demographic, anthropometric, and laboratory variables (Acronyms used: BMI body‐mass index, HIV human immunodeficiency virus, DTG dolutegravir, 3TC lamivudine, BIC bictegravir, FTC emtricitabine, TAF tenofovir alafenamide, RPV rilpivirine, ABC abacavir, DRV darunavir, DOR doravirine, TDF tenofovir disoproxil fumarate, cFT calculated free testosterone, FSH follicle‐stimulating hormone, LH luteinizing hormone, PRL prolactin hormone, PSA prostate specific antigen, SHBG sex hormone binding globulin, SD standard deviation).
**Supporting Table 2**: Results from inferential analysis of the relationship between the results of the questionnaires and the ultrasound variables (Acronyms used: PSV peak systolic velocity, EDV end diastolic velocity, IMT intima‐media thickness, IR resistive index. Level of significance: * p‐value < 0.05).
**Supporting Table 3**: Concordance between IIEF‐5 and SIEDY Scale 1 in the classification of erectile dysfunction (Acronyms used: IIEF, International Index of Erectile Function; SIEDY, Structured Interview on Erectile Dysfunction. Level of significance: * p‐value < 0.05).
**Supporting Table 4**: Prevalence of Pathological SIEDY Scales and Statistical Comparisons (Acronyms used: SIEDY, structured interview on Erectile Dysfunction. Level of significance: * p‐value < 0.05).
