# Supplementary material for: SIEDY Diagnostic Accuracy in Assessing Erectile Dysfunction in Young Men Living With HIV
Source: Andrology. 2026 Mar 9;14(7):2290–6. doi: 10.1111/andr.70215 (PMC13592048; doi:10.1111/andr.70215)
Supplement: Supplementary file 1 [file ANDR-14-2290-s001.docx]

***SUPPLEMENTARY MATERIALS***

Table S1 Demographic, anthropometric and laboratory variables (Acronyms used: BMI body-mass index, HIV human immunodeficiency virus, DTG dolutegravir, 3TC lamivudine, BIC bictegravir, FTC emtricitabine, TAF tenofovir alafenamide, RPV rilpivirine, ABC abacavir, DRV darunavir, DOR doravirine, TDF tenofovir disoproxil fumarate, cFT calculated free testosterone, FSH follicle-stimulating hormone, LH luteinizing hormone, PRL prolactin hormone, PSA prostate specific antigen, SHBG sex hormone binding globulin, SD standard deviation)

| Sample size, n (%) | 50 (100) |
| --- | --- |
| Age (years), mean (SD) | 44.4 (4.3) |
| BMI (kg/m2), mean (SD) | 26.4 (4.4) |
| Viro-immunological |  |
| Years living with HIV, mean (SD) | 13.9 (6.77) |
| CD4 nadir, mean (SD) | 285.7 (202.53) |
| HIV-RNA zenit, mean (SD) | 377,176.6 (863,099.40) |
| Undetectable viraemia, n (%) | 50 (100) |
| Current CD4 cells count, mean (SD) | 837.9 (417.57) |
| Current CD4 cells %, mean (SD) | 32.1 (12.05) |
| Current CD8 cells count, mean (SD) | 1073.4 (477.6) |
| Current CD8 cells %, mean (SD) | 40 (10.35) |
| Current CD4/CD8 ratio, mean (SD) | 0.9 (0.45) |
| Antiretroviral regimen |  |
| DTG/3TC, n (%) | 20 (40) |
| BIC/FTC/TAF, n (%) | 15 (30) |
| DTG/RPV, n (%) | 5 (10) |
| RPV/FTC/TAF, n (%) | 3 (6) |
| DTG/ABC/3TC, n (%) | 3 (6) |
| DRV/c/DTG, n (%) | 2 (4) |
| DOR/FTC/TDF, n (%) | 1 (2) |
| DRV/c/FTC/TAF, n (%) | 1 (2) |
| Comorbidities |  |
| Arterial hypertension, n (%) | 10 (20) |
| Dyslipidaemia, n (%) | 9 (18) |
| Psychiatric diseases, n (%) | 8 (16) |
| Neurological or pelvical diseases, n (%) | 4 (8) |
| Diabetes, n (%) | 4 (8) |
| Chronic kidney disease, n (%) | 2 (4) |
| Currently or previous smoking, n (%) | 15 (30) |
| Hormonal assays |  |
| Hypogonadism, n (%) | 7 (14) |
| Total testosterone (µg/L), mean (SD) | 4.4 (2.1) |
| SHBG (nmol/L), mean (SD) | 38.4 (14.5) |
| cFT (ng/dL), mean (SD) | 94.7 (29.4) |
| FSH (IU/L), mean (SD) | 6.7 (5.7) |
| LH (IU/L), mean (SD) | 7.7 (5.2) |
| PRL (µg/L), mean (SD) | 15.8 (15.4) |
| PSA (µg/L), mean (SD) | 1.2 (1.5) |

Table S2 Results from inferential analysis of the relationship between the results of the questionnaires and the ultrasound variables (Acronyms used: PSV peak systolic velocity, EDV end diastolic velocity, IMT intima-media thickness, IR resistive index. Level of significance: * p-value < 0.05)

| Variable | Questionnaire | Test Type | p-value | Significance |
| --- | --- | --- | --- | --- |
| PSV (continuous) | SIEDY scale 1 | Wilcoxon | 0,06441 |  |
| EDV (continuous) | SIEDY scale 1 | Wilcoxon | 0,1533 |  |
| IR (continuous) | SIEDY scale 1 | Wilcoxon | 0,08047 |  |
| IMT (continuous) | SIEDY scale 1 | Wilcoxon | 0,917 |  |
| PSV (continuous) | SIEDY scale 2 | Wilcoxon | 0,5108 |  |
| EDV (continuous) | SIEDY scale 2 | Wilcoxon | 0,7404 |  |
| IR (continuous) | SIEDY scale 2 | Wilcoxon | 0,425 |  |
| IMT (continuous) | SIEDY scale 2 | Wilcoxon | 0,029 | * |
| PSV (continuous) | SIEDY scale 3 | Wilcoxon | 0,2353 |  |
| EDV (continuous) | SIEDY scale 3 | Wilcoxon | 0,2686 |  |
| IR (continuous) | SIEDY scale 3 | Wilcoxon | 0,4361 |  |
| IMT (continuous) | SIEDY scale 3 | Wilcoxon | 0,042 | * |
| PSV (categorical) | SIEDY scale 1 | Fisher | 0,4361 |  |
| EDV (categorical) | SIEDY scale 1 | Chi-squared | 0,6229 |  |
| IR (categorical) | SIEDY scale 1 | Chi-squared | 0,5029 |  |
| IMT (categorical) | SIEDY scale 1 | Chi-squared | 0,791 |  |
| PSV (categorical) | SIEDY scale 2 | Fisher | 1 |  |
| EDV (categorical) | SIEDY scale 2 | Chi-squared | 0,9342 |  |
| IR (categorical) | SIEDY scale 2 | Chi-squared | 1 |  |
| IMT (categorical) | SIEDY scale 2 | Fisher | 0,1702 |  |
| PSV (categorical) | SIEDY scale 3 | Fisher | 0,1428 |  |
| EDV (categorical) | SIEDY scale 3 | Chi-squared | 0,6655 |  |
| IR (categorical) | SIEDY scale 3 | Chi-squared | 0,8044 |  |
| IMT (categorical) | SIEDY scale 3 | Chi-squared | 0,083 |  |
| PSV (continuous) | SIEDY scale 1 | Logistic Regression | 0,0672 |  |
| EDV (continuous) | SIEDY scale 1 | Logistic Regression | 0,11205 |  |
| IR (continuous) | SIEDY scale 1 | Logistic Regression | 0,0259 | * |
| IMT (continuous) | SIEDY scale 1 | Logistic Regression | 0,993 |  |
| PSV (continuous) | SIEDY scale 2 | Logistic Regression | 0,198 |  |
| EDV (continuous) | SIEDY scale 2 | Logistic Regression | 0,704 |  |
| IR (continuous) | SIEDY scale 2 | Logistic Regression | 0,235 |  |
| IMT (continuous) | SIEDY scale 2 | Logistic Regression | 0,031 | * |
| PSV (continuous) | SIEDY scale 3 | Logistic Regression | 0,381 |  |
| EDV (continuous) | SIEDY scale 3 | Logistic Regression | 0,126 |  |
| IR (continuous) | SIEDY scale 3 | Logistic Regression | 0,187 |  |
| IMT (continuous) | SIEDY scale 3 | Logistic Regression | 0,045 | * |

Table S3 Concordance between IIEF-5 and SIEDY Scale 1 in the classification of erectile dysfunction (Acronyms used: IIEF, International Index of Erectile Function; SIEDY, Structured Interview on Erectile Dysfunction. Level of significance: * p-value < 0.05)

|  | Pathological SIEDY Scale 1 | Normal SIEDY Scale 1 |
| --- | --- | --- |
| Pathological IIEF-5 | 14 (28%) | 33 (66%) |
| Normal IIEF-5 | 0 (0%) | 3 (6%) |
| Fisher’s exact test, p = 0.55  Cohen’s kappa (κ) = 0.048, p = 0.265 | | |

Table S4 Prevalence of Pathological SIEDY Scales and Statistical Comparisons (Acronyms used: SIEDY, structured interview on Erectile Dysfunction. Level of significance: * p-value < 0.05)

| SIEDY Scale | Pathological Cases, n (%) | Pairwise Comparisons  (Chi-Square with Bonferroni Correction) | Pairwise Comparisons  (McNemar Test) |
| --- | --- | --- | --- |
| Scale 1 | 14 (24%) | Scale 3 vs. Scale 1: p < 0.001 | Scale 3 vs. Scale 1: p < 0.001 |
| Scale 2 | 18 (36%) | Scale 3 vs. Scale 2: p = 0.004 | Scale 3 vs. Scale 2: p < 0.001 |
| Scale 3 | 35 (70%) | - | - |
| Chi-Square Test: X² = 20.12, df = 2, p < 0.0001  Cochran’s Q Test: Q = 14.92, df = 2, p = 0.0006 | | |  |
